# A Neuregulin-1 schizophrenia susceptibility variant causes perihippocampal fiber tract anomalies in healthy young subjects

**DOI:** 10.1002/brb3.203

**Published:** 2014-01-19

**Authors:** Thomas Nickl-Jockschat, Tony Stöcker, Axel Krug, Valentin Markov, Ruiwang Huang, Frank Schneider, Ute Habel, Simon B Eickhoff, Klaus Zerres, Markus M Nöthen, Jens Treutlein, Marcella Rietschel, Nadim Jon Shah, Tilo Kircher

**Affiliations:** 1 Department of Psychiatry, Psychotherapy and Psychosomatics, RWTH Aachen UniversityAachen, Germany; 2 Juelich Aachen Research Alliance – Translational Brain MedicineJuelich/Aachen, Germany; 3 Institute of Neurosciences and Medicine-4, Juelich Research CenterJuelich, Germany; 4 Department of Psychiatry and Psychotherapy, University of MarburgMarburg, Germany; 5 Institute of Clinical Neuroscience and Medical Psychology, Heinrich Heine UniversityDüsseldorf, Germany; 6 Department of Neuroscience und Medicine, INM-1, Research Center JülichJülich, Germany; 7 Institute of Human Genetics, RWTH Aachen UniversityAachen, Germany; 8 Department of Genomics, Life and Brain Center, University of BonnBonn, Germany; 9 Department of Genetic Epidemiology in Psychiatry, Central Institute of Mental HealthMannheim, Germany; 10 Department of Neurology, RWTH Aachen UniversityAachen, Germany

**Keywords:** Anatomic connectivity, brain, DTI, gene, hippocampus, MRI, Neuregulin-1

## Abstract

**Background:** Changes in fiber tract architecture have gained attention as a potentially important aspect of schizophrenia neuropathology. Although the exact pathogenesis of these abnormalities yet remains to be elucidated, a genetic component is highly likely. Neuregulin-1 (*NRG1*) is one of the best-validated schizophrenia susceptibility genes. We here report the impact of the Neuregulin-1 rs35753505 variant on white matter structure in healthy young individuals with no family history of psychosis. **Methods:** We compared fractional anisotropy in 54 subjects that were either homozygous for the risk C allele carriers (*n* = 31) for rs35753505 or homozygous for the T allele (n = 23) using diffusion tensor imaging with 3T. Tract-Based Spatial Statistics (TBSS), a method especially developed for diffusion data analysis, was used to improve white matter registration and to focus the statistical analysis to major fiber tracts. **Results:** Statistical analysis showed that homozygous risk C allele carriers featured elevated fractional anisotropy (FA) in the right perihippocampal region and the white matter proximate to the left area 4p as well as the right hemisphere of the cerebellum. We found three clusters of reduced FA values in homozygous C allele carriers: in the left superior parietal region, the right prefrontal white matter and in the deep white matter of the left frontal lobe. **Conclusion:** Our results highlight the importance of Neuregulin-1 for structural connectivity of the right medial temporal lobe. This finding is in line with well known neuropathological findings in this region in patients with schizophrenia.

## Introduction

The schizophrenias are a group of—most likely pathophysiologically and etiologically heterogeneous—disorders that go along with progressively disabling deficits in cognition and behavior. Although the exact etiopathogenesis yet remains to be fully elucidated, current literature suggests the view of largely genetically determined disorders with changes in brain structure and function (Meyer-Lindenberg 2010). On an anatomical level, structural magnetic resonance imaging (MRI) studies have yielded compelling evidence for gray matter reductions in fronto-temporo-thalamic circuits. Remarkably, the affected brain regions are functionally associated with reward, affective processes and language functions, that is, neurophysiological functions that are altered as hallmarks of schizophrenia psychopathology (Nickl-Jockschat et al. 2011). These findings strongly support the hypothesis that these brain structure changes are closely linked to schizophrenia symptomatology (Kircher et al. 2001).

Dysfunctional connectivity and altered white matter structure have been repeatedly taken into focus as a key pathophysiology of schizophrenia (Harrison and Weinberger 2005; Schmitt et al. 2011). Consequently, a growing number of diffusion tensor imaging (DTI) studies aimed to identify white matter abnormalities in schizophrenia patients. Although the number of studies still is comparatively scarce and findings are heterogeneous, convergent evidence suggests abnormal white matter properties especially in the left frontal and temporal lobe (Ellison-Wright and Bullmore 2009). Intriguingly, it has been argued that heterogeneous white matter changes might explain clinical differences between patient populations, for example, those with and without auditory hallucinations (Hubl et al. 2004).

One of the most commonly used DTI indices is fractional anisotropy (FA) (Basser and Pierpaoli 1996). FA is a scalar (between 0 and 1) that describes the degree of directionality of the diffusion in a particular voxel. An FA of zero indicates that diffusion is the same, that is, equally (un-) restricted, in all directions. At the other end of the scala, a value of one indicates that diffusion is only present in one particular direction (and fully restricted in the other ones). Since the diffusion of water in brain tissue is restricted by the coherence of the fiber tracts (Ono et al. 1995), structural fiber integrity, their diameter and packing density (Ono et al. 1995), and by myelination (Sakuma et al. 1991; Gulani et al. 2001), proxy conclusions about white matter microstructure can be drawn from a FA values that quantitate how strongly directional the local diffusion structure is.

Various studies have examined the heritability of FA in healthy subjects (Brouwer et al. 2010; Chiang et al. 2011; Kochunov et al. 2011; Jahanshad et al. 2013). A recent meta-analysis of the ENIGMA Consortium using high-resolution FA images from multiple imaging sites across North America, Australia, and Europe found high heritability for almost all fiber tracts across and within the studied cohorts (Jahanshad et al. 2013). Thus, at least in healthy subjects, white matter properties reflected in FA seems to be strongly influenced by genetics.

Healthy genetic relatives of schizophrenia patients have also been shown to display altered FA values compared with controls with no family history of psychosis in brain regions that exhibited altered FA in schizophrenia patients (Camchong et al. 2009; Knöchel et al. 2012). These findings corroborate a genetic influence on FA changes and highlight the clinical importance of studies on the association between schizophrenia susceptibility gene and FA changes. Functional genomic analyses moreover emphasize the involvement of schizophrenia susceptibility genes in synaptic and neuronal plasticity (Ayalew et al. 2012). Consequently, a genetic imaging approach to investigate associations between risk gene variants and white matter anomalies appears as a promising strategy to shed light on the underlying mechanisms of anatomical dysconnectivity.

The Neuregulin-1 (NRG1) gene is an interesting candidate in this context. It is assumed that mutations in the *NRG1* gene may lead to functional changes which, mainly in the vulnerable phases of embryonic development but also in the mature brain, may disturb neuronal development and plasticity, thus decisively contributing to the pathogenesis of mental disorders (Harrison and Weinberger 2005). The mature protein exerts its influence on these functions by binding to ErbB receptors 3 and 4. Each of these receptors can—after activation—heterodimerize with ErbB2 following a ligand-activated conformational change, leading in consequence to the activation of its intracellular downstream signaling pathways (Burgess et al. 2003). Stefansson et al. (2002) were the first to report about an association between schizophrenia and a specific NRG1 haplotype. Despite inconsistent findings, the latter, which is called Icelandic Haplotype, (HapICE) could be confirmed through meta-analyses (Li et al. 2006; Ayalew et al. 2012). The risk conferred by the HapICE haplotype has been attributed to an increase in expression level of type III NRG1, which is the isoform being most abundant in the brain (Weickert et al. 2012). Expression of Nrg1 type III has been detected in both, developing and adult brains of rodents (Bare et al. 2011) and has been implicated in determination in the extent of myelination, as brains of mice haploinsufficient for type III Nrg1 have been found to be hypomyelinated (Taveggia et al. 2008).

The original core haplotype consisted of five single-nucleotide polymorphisms (SNPs) and two microsatellite markers. Of all studied markers of the NRG1 genomic region, rs35753505, which is located in the 5′-flanking region of NRG1, is the most commonly reported single marker. Even though some authors found strong associations of rs35753505 with schizophrenia, others failed to do so (Li et al. 2006; cf. O′Donovan et al. 2008). It also needs to be noted that recent genome-wide association studies did not find a significant link of NRG1 rs35753505 to schizophrenia (cf. Stefansson et al. 2009). NRG1 rs35753505 nevertheless has the great advantage that it is one of the first SNPs that has been shown to be associated with schizophrenia (Stefansson et al. 2002), and, therefore, studies have repeatedly aimed to elucidate the biological functions of both NRG1 and rs35753505. However, results especially of imaging genetics studies often are contradictory and therefore demand replication with sound methodical approaches. Moreover, a meta-analysis found NRG1 as one of the most consistent genes to be reported in schizophrenia (Ayalew et al. 2012), underlining the role of NRG1 as schizophrenia susceptibility gene.

Since some of the functions of NRG1 influence neuronal migration and myelinisation, possible effects of *NRG1* variants on anatomical connectivity have been investigated by two DTI-based studies (McIntosh et al. 2007; Winterer et al. 2008). A study on the rs6994992 variant of the NRG1 gene reported reduced white matter integrity in the anterior limb of the internal capsule (McIntosh et al. 2007), the second investigated the effects of the rs35753505 SNP and found effects on the FA in medial frontal white matter to be associated with NRG1 variance (Winterer et al. 2008).

However, both publications reported rather discrete changes in anatomical connectivity. Thus, the use of a method with a highly precise alignment algorithm seems pivotal in imaging genetics studies, especially during the analysis of diffusion imaging-derived data sets.

Both of the studies on NRG1 used conventional VBM-style approaches for their analyses. Conventional VBM-style whole-brain approaches for multisubject FA images have been criticized for alignment (Simon et al. 2005; Vangberg et al. 2006) and smoothing issues (Jones et al. 2005). The Tract-Based Spatial Statistics (TBSS) approach addresses both of these problems by application of an initial approximate nonlinear registration, followed by the projection of the FA values onto an alignment invariant tract representation, the “mean FA skeleton” (Smith et al. 2006). The mean FA skeleton is generated in a fully automatized procedure, in which first the voxels with the regionally highest FA values are identified and then the centers of the tracts are determined by local center-of-gravity calculation. These steps are intended to enhance alignment and therefore increase sensitivity and interpretability of DTI data.

Readdressing the heterogeneous results of previous studies on *NRG1* effects on and anatomical connectivity, we thus employed this more appropriate approach to investigate the effects of the NRG1 rs35753505 variant on local FA values in 54 healthy young subjects. Since we expected genotype effects most pronounced in homozygous allele carriers, we only included subjects that were – after initial genotyping – homozygous risk (or non-risk) allele carriers for this polymorphisms, while not considering heterozygous allele carriers in this study.

## Methods

### Subjects

The study protocol was approved by the local ethics committee of the University Hospital Aachen. Subjects were recruited from RWTH Aachen University students and by advertisements in local newspapers. The inclusion criteria were as follows: age 18–55 years old, no psychiatric disorder according to ICD-10, and an absence of a family history for psychiatric disorders in first degree relatives. All subjects were of Western-or Middle European descent. Fifty-four subjects (34 males, 20 females) underwent DTI after genotyping for the *NRG1* rs35753505 variant. The subjects had a mean age of 22.9 years (SD = 2.8), were right handed (as tested with the Edinburgh Laterality Scale), and had 15.6 (2.3) years of education. Their fathers were educated for 16.0 (4.4) and their mothers for 14.4 (4.3) years on average. Mean intelligence quotient (IQ) was 112.1 (12.2). After a complete description of the procedure, subjects provided written informed consent to participate in the study (cf. Krug et al. 2008a; Kircher et al. 2009a). Blood was taken from a vein of each subject's arm.

### Genotyping

The rs35753505 was genotyped using Applied Biosystems 7900HT Fast Real-Time polymerase chain reaction (PCR) System and TaqMan-probes designed by Applied Biosystems (Foster City, CA). Primers and VIC/FAM-probe sequences for rs35753505 detection were as follows: Forward-5′-TTTAAGGCATCAGTTTTCAATAGCTTTTTTATGT-3′; Reverse-5′-AGACAGATGTCTCAAGAGACTGGAA-3′;5′-VIC-CATGTATCTTTATTTT**G**CCAAAT-3′; 5′-FAM-CATGTATCTTTATTTT**A**CCAAAT-3′. Sequence information was obtained from the homepage of deCODE Genetics (http://decode.com/nrg1/markers/SNPS.htm). Replication of 15% of the sample showed no differences in genotypes.

### Image acquisition

Imaging was performed on a 3-Tesla Trio MR scanner (Siemens Medical Systems, Erlangen, Germany) in the Institute of Neuroscience and Biophysics—Medicine, Research Center Jülich. Subjects for the scans were chosen due to their genotype. Head movements were minimized by immobilizing the head during the scanning procedure using foam cushions. Images were acquired with a diffusion-weighted (DW) double spin-echo echo planar imaging sequence (echo time 89 msec; 1.8 mm isotropic resolution). A 12-channel phased-array coil was used and the sequence utilized twofold acceleration with the GRAPPA parallel imaging technique (Griswold et al. 2002). Sixty different gradient directions distributed over the unit sphere according to the Jones-scheme were acquired with a *b*-value of 800 sec/mm^2^, in addition seven interleaved acquisitions of non-DW images (*b* = 0). The protocol was acquired four times and, after individual motion correction, the DW images were averaged to increase the signal-to-noise-ratio. In addition, an anatomical T1-weighted magnetization prepared rapid gradient echo sequence was acquired (1 mm isotropic resolution).

### Image preprocessing

We followed the standard protocol by Smith and colleagues (Smith et al. 2007). First, data sets were corrected for head motion and eddy currents. Then, a diffusion tensor model was fit to the set of diffusion-weighted images, before calculating FA maps for each subject. All FA images were visually checked for artefacts, intensity range problems, and general data quality.

### TBSS analysis

After visual assessment, we used the FSL TBSS scripts (http://www.fmrib.ox.ac.uk/fsl/tbss) on the individual FA maps (Smith et al. 2006, 2007). All individual FA maps were nonlinearly registered to each other to determine the “most typical” subject of each group. After identification of the “most typical” subject as the target, all other FA images were aligned to it and then transformed into 1 × 1 × 1 mm^3^ MNI152 space. All subsequent processing was carried out using this space and resolution. The transformed images were averaged to create a mean FA image, which was then fed into the tract skeleton generation, resulting in an FA skeleton aiming to represent all fiber tracts common to all subjects included in the study. To restrict further analysis to the white matter, a skeleton threshold of FA > 0.2 was applied (Smith et al. 2007). Then, the nearest local FA maxima of each individual FA image were projected onto the mean FA skeleton.

This process of registration helps to increase sensitivity and interpretability of results yielded by DT imaging. For example, ventricular enlargement caused by a pathophysiological process can notably mislead the interpretation of the results of a voxel-based voxel-based morphometry (VBM)-style DTI analysis (Smith et al. 2007). The step of projecting individual FA maps onto a mean FA skeleton helps to confine the effect of cross-spatial subject variability that remains after the nonlinear registration. Especially in studies, in which group differences are expected to be small such as imaging genetics approaches, TBSS is valuable to limit artefacts and provide more precise results.

### Statistical analysis

Comparison between the homozygotic wild type T allele carriers and homozygotic risk C allele carriers was performed by means of a two-sample non-parametric *t*-test on the FA values along the tract skeleton. Statistical inference was determined using a permutation-based approach (Nichols and Holmes 2002) with 6400 permutations to establish a null distribution of differences and derive nonparametric *P*-values for the group comparison. We used the “randomize”—tool with TFCE (threshold free cluster enhancement)—option as implemented in FSL. To control for gender effects, subjects' sex was included as a covariate of no interest into the statistical model. The t-maps were then thresholded at *P* (uncorr.) <0.001 and projected onto the mean FA skeleton for visualization.

For a more detailed anatomical analysis, we used the Anatomy Toolbox (Eickhoff et al. 2005, 2007) to compare the localization of the obtained significant effects to myeloarchitectonical probability maps derived from the histological analysis of 10 human postmortem brains (Bürgel et al. 2006), spatially normalized into the MNI reference space. These maps quantify how often a particular tract has been found at each position of the white matter in the reference space. They were then combined into a Maximum Probability Map, which is a summary map of the probabilistic information. It is based on the idea of attributing each voxel of the reference space to the most likely myeloarchitectonically defined fiber-tract at this position. Maximum Probability Maps thus allow the definition of nonoverlapping representations of all areas from a set of inevitably overlapping probabilistic maps (Eickhoff et al. 2006).

## Results

### Demographics

The images of 54 subjects (34 males, 20 females) were included in our TBSS analysis. The homozygous wild-type group consisted of 31 subjects (20 males, 11 females), the homozygous risk allele carrier group consisted of 23 subjects (14 males, nine females). Mean age in the wild-type group was 23.1 years (SD: 3.2 years), in the homozygous risk allele carrier group 22.6 (SD: 2.2 years). Both groups did not differ significantly in mean age (*P* = 0.5) or gender (*P* = 0.503). Also mean IQ (wild type: 111.7 [SD: 11.8], risk type 112.6 [SD: 13.0]) was not significantly different in both groups (*P* = 0.8).

### Impact of the NRG1 genotype on fiber tract integrity

**Figure 1:**
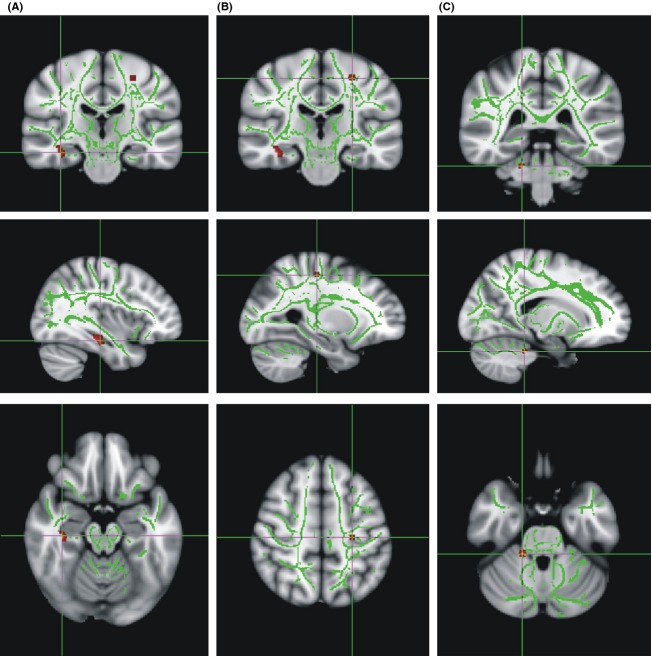
Clusters of increased FA values in homozygous NRG1 rs35753505 risk C allele carriers. Clusters were located in the right perihippocampal region (38, −29, −10) (A), the white matter proximate to the left area 4p (−26, −27, 57) (B) and the right hemisphere of the cerebellum (18, −40, −24) (C). Major fiber tracts as determined by TBSS are shown in green, while the red clusters indicate increased FA values in NRG1 risk C allele carriers (*P* [uncorr.] <0.001). FA, fractional anisotropy; NRG, Neuregulin; TBSS, Tract-Based Spatial Statistics.

The statistical analysis revealed three clusters higher FA values in homozygous C allele carriers. The largest of these clusters was located in the right peri-hippocampal region (38, −29, −10, *k* = 504), while one cluster was situated in the white matter proximate to the left area 4p (−26, −27, 57, *k* = 123). The smallest cluster was located in the right hemisphere of the cerebellum (18, −40, −24, *k* = 114) (Fig. 1).

**Figure 2:**
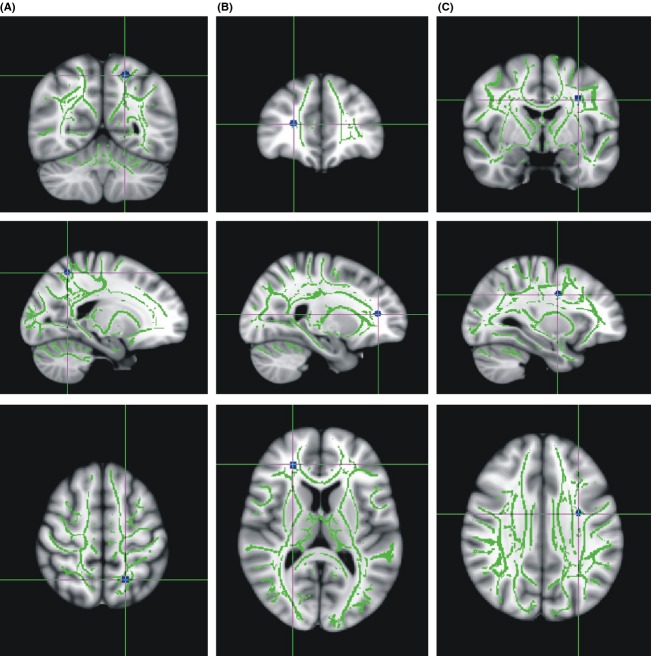
Clusters of reduced FA values in homozygous C allele carriers. They were located in the left superior parietal region (−19, −60, 61) (A), the right prefrontal white matter (24, 35, 17) (B) and in the deep white matter of the left frontal lobe (−30, −7, 39). Major fiber tracts as determined by TBSS are shown in green, while blue clusters indicate decreased FA values in NRG1 risk C allele carriers (*P* [uncorr.] <0.001). FA, fractional anisotropy; TBSS, Tract-Based Spatial Statistics; NRG, Neuregulin.

We also detected three clusters of reduced FA values in homozygous C allele carriers. One of these clusters was located in the left superior parietal region (−19, −60, 61, *k* = 152). Another cluster was located in the right prefrontal white matter (24, 35, 17, *k* = 152). A third cluster was situated in the deep white matter of the left frontal lobe (−30, −7, 39, *k* = 123) (Fig. 2).

## Discussion

NRG1 has been shown to induce neurite outgrowth in different neuronal populations (Rieff et al. 1999; Gerecke et al. 2006). Moreover, a role in axonal guidance has also been highlighted. In a study using a mouse model, Lopez-Bendito and colleagues showed that the proper outgrowth of thalamocortical axons requires so-called “corridor cells.” These corridor cells express high levels of cysteine-rich-domain-containing NRG1 (CDR-NRG1, synonymously NRG1 type III) that is thought to activate ErbB4-dependent signaling in TCAs, allowing further growth passing through the developing diencephalon. Soluble Ig-NRG1 from the ventral and lateral pallidum serves as long-range attractant inducing TCA migration through the dorsal striatum and into the cortex (López-Bendito et al. 2006). Thus, there is evidence that dysfunctional NRG1 signaling during embryonic development plays a role in the pathogenesis of fiber tract anomalies. Various studies have shown a functional impact of NRG1 isoforms on the hippocampal formation, thus suggesting potential mechanisms causing changes of anatomical connectivity in this region. Studies using recombinant Neuregulin-1 on murine hippocampal slides suggest that NRG1/ErbB-dependent signalling suppresses both the induction and suppression of long-term potentiation (LTP) (Mei and Xiong 2008). Remarkably, data derived from a knock-out mouse model with a heterozygous NRG1 deletion indicated that at least theta-burst-induced LTP was enhanced, not suppressed, after low-dose application of recombinant *Nrg1*, while higher concentrations reversed this effect (Bjarnadottir et al. 2007). These findings let to the hypothesis that decreased NRG1 levels during neurodevelopment lead to changes in NRG1 signaling-dependent effects on LTP. Consequently, consistent alterations of neuronal activity and reactivity to NRG1 signalling might lead to changes in the shaping of the perihippocampal fibers.

Changes in brain structure (Shenton et al. 2001; Glahn et al. 2008; Fornito et al. 2009; Nickl-Jockschat et al. 2011) and fiber tract architecture (Ellison-Wright and Bullmore 2009) of the medial temporal lobe have been consistently found in schizophrenia patients. Although it has to be noted that most studies found effects lateralized to the dominant hemisphere (Ellison-Wright and Bullmore 2009; Nickl-Jockschat et al. 2011), several DTI-based studies report reduced FA values to be pronounced in the right medial temporal lobe in schizophrenia patients (Schlösser et al. 2007; Phillips et al. 2009). Moreover, gray matter alterations of the medial temporal lobe have been described for NRG1 risk variant carriers. In a study on schizophrenia patients and their nonaffected family members, both patients and relatives carrying the HapICE had smaller relative hippocampal volumes than wild types (Gruber et al. 2008). Since the rs35753505 is the most commonly reported single marker of the HapICE, these findings directly relate to the current results. Functional imaging studies furthermore highlight the pathophysiological relevance of these anatomical findings. Schizophrenia patients have been reported to exhibit less functional lateralization in the temporal lobes (Sommer et al. 2001, 2003). Moreover, hippocampal dysfunction has been repeatedly observed in schizophrenia patients, for example, during free verbal association (Kircher et al. 2008).

Consequently, the *NRG1* genotype-dependent perihippocampal FA changes found in our study could be an anatomical marker for increased vulnerability, in particular when considering findings on the Dysbindin (*DTNBP1*) rs1018381 schizophrenia susceptibility variant, which was associated with FA reductions in the right perihippocampal region (Nickl-Jockschat et al. 2012). This result supports the hypothesis that the right perihippocampal white matter could be an anatomical substrate of genetic liability for schizophrenia.

To our surprise, carriers of the rs35753505 risk C allele showed elevated FA values in the right perihippocampal region. Since the C allele is associated with schizophrenia (Li et al. 2006), we expected rs35753505 C allele carriers to exhibit reduced FA values in brain regions associated with schizophrenia.

However, schizophrenia is usually seen as a polygenic disorder (Insel 2010; McClellan and King 2010). NRG1/ErbB-dependent signaling is involved in a multitude of biological functions that are key factors in schizophrenia pathophysiology (Mei and Xiong 2008). An interaction with other schizophrenia susceptibility genes variants therefore seems highly likely. Such an interaction with Neuregulin-1 has been proposed, for example, for disrupted in schizophrenia 1 (DISC1). Moreover, both NRG1 and DISC1 interact with growth factor receptor-bound protein 2 (Grb2), an adaptor protein located in the postsynaptic densities (Jaaro-Peled et al. 2009). Moreover, NRG1 has been shown to induce phosphorylation of Akt (Guo et al. 2010). Akt is a central hub in various signaling pathways and involved in schizophrenia pathophysiology (Zheng et al. 2012). Consequently, a different intracellular environment might lead to differential effects on axonal outgrowth and integrity.

We found another cluster of elevated FA in the white matter proximate to the area 4p. Area 4p is a cytoarchitectonically defined subdivision of the human primary motor cortex (Geyer et al. 1996). We therefore interpreted the changes as most likely attributable to the internal capsule. Abnormalities of white matter density of the internal capsule have been described previously in schizophrenia patients (Zhou et al. 2003; McIntosh et al. 2005). Also, a study on the *NRG1* rs6994992 variant reported reduced white matter integrity in the anterior limb of the internal capsule (McIntosh et al. 2007). While it would be premature to relate these changes to pathophysiological processes it is still noteworthy that there are effects of two different NRG1 variants on fiber tract integrity of the internal capsule. A third cluster of elevated FA in C allele carriers was found in the right hemisphere of the cerebellum. Lateral cerebellar dysfunction has been proposed to lead to deficits of higher cognitive functions (Schmahmann and Sherman 1998) and as a contributor to schizophrenia pathophysiology (Andreasen et al. 1998). DTI studies found cerebellar FA reductions in schizophrenia patients (Okugawa et al. 2006; Kyriakopoulos et al. 2008). In contrast to these findings, our results indicated higher cerebellar FA values similar to what has previously been shown for a *DTNBP1* risk variant (Nickl-Jockschat et al. 2012). This convergence of results suggests a role for cerebellar fiber tract integrity in genetic liability to psychosis.

In contrast to our initial hypothesis, FA reductions were less prominent than FA increases in risk C allele carriers. The largest cluster was situated in the left superior parietal region. FA changes in the parietal lobe have been less frequently reported in schizophrenia than abnormalities of frontal and temporal white matter. Nevertheless, alterations of fronto-parietal anatomical connectivity have been described in subjects with deficit schizophrenia (Rowland et al. 2009) although on the right hemisphere.

Consistent with a previous study on the rs35753505 variant (Winterer et al. 2008), we found FA reductions in C allele homozygotes in the frontal lobe. Disturbances of frontal lobe white matter integrity are among the best reproduced findings in schizophrenia patients (Ellison-Wright and Bullmore 2009). This finding relates well to recent functional genomics imaging studies in an overlapping cohort showing that *NRG1* rs35753505 genotype status influenced frontal brain activation during working memory (Krug et al. 2008b) and verbal fluency tasks (Kircher et al. 2009b). Also during episodic memory encoding, the *NRG1* rs35753505 genotype modulated frontal brain activation (Krug et al. 2010). Changes in frontal brain activation were not unidirectionally influenced by C allele carrier status. While the number of C alleles were correlated with increased frontal activations during a working memory task and episodic memory encoding (Krug et al. 2008b, 2010), the opposite was found during a verbal fluency task (Kircher et al. 2009b). Since there were no behavioural changes due to genotype during the two firstly mentioned tasks (Krug et al. 2008b, 2010), but verbal fluency decreased with C allele frequency (Kircher et al. 2009), increased BOLD responses were interpreted as a compensatory mechanism. The changes in frontal fiber tract integrity found here might well be the anatomical basis for these functional alterations.

While decreases in frontal FA have been described in NRG1 rs35753505 risk type carriers, the overall pattern of changes found in our data set is markedly differed from that of a previously published study on this SNP (Winterer et al. 2008). Especially increases in FA were not reported. In contrast, the largest cluster in our study was found in the right perihippocampal region and indicated higher FA in homozygous risk allele carriers. There are several possible explanations for these differences. First of all, while in the study by Winterer and colleagues homo-and heterozygote C allele carriers were compared to T allele homozygotes for their whole-brain analyses, we here focused on homozygous C and T allele carriers. As neuroanatomical changes should be most pronounced in homozygotes, this approach may have yielded a higher sensitivity to subtle effects. Adding to this effect, we here used the TBSS algorithm. This method was specifically developed for the analysis of diffusion imaging data (Smith et al. 2006). Given the methodical problems of conventional VBM-style whole-brain approaches for multisubject FA images with regard to alignment (Simon et al. 2005; Vangberg et al. 2006) and smoothing (Jones et al. 2005), TBSS provides an optimized solution for diffusion data analysis. Previous studies demonstrated that the application of TBSS is especially suitable for imaging genetics studies, where between-genotype differences often are small and therefore precise alignment is critical to avoid false positive or false negative findings (Nickl-Jockschat et al. 2012). Especially in a brain region such as the medial temporal lobe, where a variety of gray and white matter structures are located close to each other, misalignment can be a critical problem. Moreover, interindividual variance is comparatively high in the neuroanatomy of the medial temporal lobe. The solution of these problems is a largely optimized alignment procedure as provided by TBSS. Consequently, our finding of elevated FA values in the right perihippocampal region might be due to improvements in data processing, in specific by using the TBSS algorithm.

Both FA increases and decreases were found in NRG1 rs35753505 risk type carriers. Given that there is still an open discussion on the microstructural correlates of FA, there are several possible scenarios for the link between the genetic variations and diffusion indices. Fundamentally, FA reflects how strongly the local diffusion of water molecules is biased in a given direction. In the cerebral white matter, major neuroanatomical influences on FA that are currently discussed are the coherence of fiber tracts (Ono et al. 1995), structural fiber integrity, their diameter and packing density (Ono et al. 1995), and by myelination (Sakuma et al. 1991; Gulani et al. 2001). Importantly, all of these may be, at least indirectly, related to NRG1 effects. In knockout mice, NRG1 has been shown to influence hippocampal LTP. Animals displayed impaired theta burst-induced LTP compared to wild types, but deficits could be rescued by the application of recombinant NRG1. Remarkably, low to medium doses of recombinant doses of recombinant NRG1 led to an increase of LTP in mutant mice, while higher doses suppressed LTP (Bjarnadottir et al. 2007). These findings strongly support the idea that NRG1 influences synaptic plasticity in a dose-dependent way. Given the fact that NRG1 expression varies between brain regions (Bare et al. 2011), differential effects of the NRG1 rs35753505 mutation on synaptic plasticity in different neuronal populations seem likely. Changes in synaptic plasticity in turn are likely to result in downstream effects on axonal trophics and ultimately structure. These changes in axonal structure in turn could give rise to differences in FA. Given the complex, dose-dependent and regional effects of NRG1 on synaptic function and thus probably axonal properties, it may in fact not surprise that both increases and decreases in FA were observed.

Myelination is considered another factor of relevant impact on FA values (Sakuma et al. 1991; Gulani et al. 2001) that has been shown to be influenced at least by NRG1 type III (cf. Taveggia et al. 2008). Unfortunately, to the best of our knowledge, there is currently no experimental data available on dose-dependent effects of NRG1 on myelination. It is nevertheless tempting to hypothesize that not only synaptic plasticity but also myelination might be differentially influenced in different brain regions by the NRG1 rs35753505 mutation.

Finally, NRG1 has also been shown to influence axonal migration during early brain development. An intricate interplay of different NRG1 isoforms is crucial for a proper migration (López-Bendito et al. 2006). We would expect this aspect to have the most fundamental and differential effect on FA values, as potentially altered migration patterns may substantially influence local fiber density and organization in NRG1 rs35753505 risk allele carriers.

Given the complex biological functions of NRG1, an interaction between the different mechanisms alluded to above seems to be the most likely mechanistic underpinning of the bidirectional FA changes found by our study. However, our results do not allow a definite conclusion about the microstructural correlates, which need to be addressed by combining microstructural and diffusion analyses in the same brains (of model animals).

There are several limitations in this study. First, the changes in brain structure we found were rather discrete. It has to be considered however that we examined healthy young subjects with a high level of intellectual functioning and without history of psychiatric disorder in first degree relatives.

Moreover, it needs to be pointed out that our results were not corrected for multiple comparisons. However, the significance threshold was comparable to the study of Winterer et al. (2008) and even surpassed the statistical significance of a study published by McIntosh et al. (2007). Nevertheless, this is a major limitation of our study.

The fact that these results were not so pronounced as to survive correction for multiple comparisons raises the problem of false positive findings. Underpowered studies due to small sample sizes can be a critical factor in the generation of false positive results. This becomes even more problematic when the effects studied are rather subtle. Given the rather low odds ratios of many schizophrenia susceptibility gene variants, also sample sizes that are usually regarded as sufficient in structural imaging studies can thus be relatively small and entail a potential danger false positive findings. The balance between controlling type I and type II errors is indeed a pertinent problem in neuroimaging. Much of this is related to the fact that, in particular at currently employed finer spatial resolution, the number of assessed voxels and hence the number of parallel tests are extremely high (up to several hundreds of thousands). This renders correction for multiple comparisons very conservative and biased toward false negative findings. It also needs to be pointed out that due to the indirect nature of the diffusion MR signal as a proxy measure for fiber tract integrity and in particular the usually “relatively” low sample size (including random effects from sampling) limit the capacity to completely exclude false positive findings even despite conservative thresholding. Conversely, more liberal thresholds obviously entail the increased danger of identifying random noise in the data, for example, due to the sampling of the subjects, as true effects. Importantly, however, such effects should not be reproducible across studies. In other words, even highly conservative inference, bringing with it a high danger of false negatives, may not necessarily protect against effects due to random sampling of a relatively small group from the underlying population. Importantly, these effects would not be false positives in the statistical sense (as they are “real” for the data given), but still would reflect findings that are not reproducible in further studies from the underlying general population. One potential way to overcome this predicament not only in diffusion analysis but also in neuroimaging per se is the focus on consistency of findings across studies (Eickhoff et al. 2009, 2012). This approach provides an important balance between sensitivity (though potentially at the expense of false positives) in the individual experiments and specificity through converging evidence. Following this line, image-and coordinate-based meta-analyses as well as data-sharing approaches are currently becoming increasingly important in imaging neuroscience. We would argue that this approach may provide a better capture of true effects in the underlying population as more conservative thresholding in an individual study. Nevertheless, we acknowledge that a formal correction for type II error within each individual study is highly advantageous if the effects are robust enough.

Although our sample consisted of populations that could potentially differ in the frequency of the risk allele due to a different ethnical background, we did not genotype the sample for ancestry informative markers. Consequently, effects of ancestry could have led to additional variance in our data.

We included both male and female subjects in this study. Although gender was used as a covariate, a direct comparison between male and female T allele carriers would be interesting. However, the number of subjects included does not allow such a comparison. Future studies enrolling larger populations should also focus on gender-specific effects.

In summary, we found both clusters of elevated and reduced FA in NRG1 rs35753505 C allele risk type carriers. Changes were most pronounced in the right perihippocampal region, where risk type carriers showed elevated FA values. The structural alterations described might in part be responsible for differences in BOLD response found by functional imaging studies in a largely overlapping population (Krug et al. 2008b, 2010; Kircher et al. 2009b).
